# Advanced Surface Treatment Technologies for Metallic Alloys

**DOI:** 10.3390/ma15041464

**Published:** 2022-02-16

**Authors:** Petrica Vizureanu

**Affiliations:** 1 Faculty of Material Science and Engineering, Gheorghe Asachi Technical University of Iasi, 41 D, Mangeron Bulevardul, 700050 Iasi, Romania; peviz@tuiasi.ro; 2 Materials Science and Engineering Department, Technical Sciences Academy of Romania, Bulevardul Dacia 26, 030167 București, Romania

The main objective of this Special Issue was to publish outstanding papers presenting cutting-edge research in the field of surface treatment technologies for metallic alloys and their understanding.

At present, more and more surface treatment procedures and technologies are available next to advanced characterization techniques. The Special Issue managed to gather several outstanding articles in a broad field, from ferrous alloys (steel, cast iron) or magnetite nanoparticles/nanowires to non-ferrous alloys or amorphous materials.

In this way, the focus was on steel in ductile iron, materials used in numerous industrial sectors. The results of an analysis of the influence of abrasive impact erosion on surface and properties of DC03/1.0347, DC04/1.0338, DC05/1.0312, and DD14/1.0389 deep drawing steels indicate that the erosive impact may cause a significant grain refinement of the microstructure of the surfaces of the investigated materials [1]. Moreover, large amounts of heat released during erosive impact may cause the material phase changes. This research expands the knowledge on specific mechanisms taking place during sandblasting and their influence on the properties of deep drawing steels and their wear behavior.

Additionally, for improving the corrosion resistance of carbon steel, used in carabiners manufacturing, three different insoluble phosphate layers were deposited on the samples’ surface. In this way, the overall results show that all types of deposited layers increase the corrosion resistance of C45 steel [2]. The experimental results revealed that the samples coated with a phosphate layer obtained by immersion in the zinc-based phosphate solution possess the highest corrosion resistance among the phosphate samples. Hot-dip aluminizing of ferrite nodular cast iron was carried out after treating liquid aluminum with different electrical pulse parameters [3]. The thickness of the alloy layer decreased, and the compactness and the micro hardness increased, so the electric pulse had a certain inhibition on the formation of the alloy layer. The growth kinetics of the alloy layer showed that the rate-time index decreased from 0.60 for the conventional sample to 0.38 for the electric pulse treated sample. The growth of the alloy layer was controlled by a diffusion and interface reaction, but only by diffusion. The AC impedance and polarization curves of the coating showed that the corrosion resistance of the hot-dip coating on nodular cast iron was improved by electric pulse treatment and the thickness and compactness of the coating surface layer increased.

Another focused on new copper matrix nanocomposites reinforced with magnetite nanoparticles that were developed using powder metallurgy [4]. These Cu-xFe_3_O_4_ nanocomposites have good cold-rolling deformability, Vickers micro-hardness, and thermal and magnetic properties that make them suitable for electronical applications.

The design of metastable retained austenite is the key issue to obtain nano bainitic steel with high strength and toughness. Nanostructured Fe-based bainitic coatings were fabricated using laser cladding and following isothermal heat treatment [5]. The volume fractions of each identified phase and microstructure morphology of the coatings were determined by the interdendritic micro-segregation and isothermal temperatures. The stability of the blocky retained austenite distributed at the interdendritic area was lower than that of film and island-like morphology. This phenomenon contributed to the ductile and tough nano bainitic coatings with tunable mechanical properties.

A simple and inexpensive thermal oxidation process was performed to synthesize gallium oxide (Ga_2_O_3_) nanowires using Ag thin film as a catalyst at 800 °C and 1000 °C to understand the effect of the silver catalyst on the nanowire growth [6]. Based on the characterization results (XRD, SEM, FIB, XPS and STEM), the Ag thin film produces Ag nanoparticles at high temperatures and enhances the reaction between oxygen and gallium, contributing to denser and longer Ga_2_O_3_ nanowires compared to those grown without silver catalyst.

Non-ferrous alloys as a category of materials was treated with advanced and modern technologies. Laser penetration welding of magnesium alloys and pure titanium TA2 with unequal thickness was presented. Mg base metal with different Al content (AZ31B, AZ61A, AZ91D) was used to investigate the influence of Al element in microstructure and mechanical properties of Mg/Ti dissimilar joints [7]. The results revealed that the change in Mg base metal did not influence the weld appearance of the joints. The highest enrichment of Al element was obtained and its fraction reached 19.31 at% at the AZ91/TA2 interface. The chemical potential gradient of Al from AZ91 to Ti alloy was higher than that from the other two base metals based on thermodynamic calculation. Moreover, the mechanical properties of the Al-Mg alloy can be enhanced by adding metallic elements, but a continuous distribution of precipitates at grain boundaries leads to intergranular corrosion during sensitization treatment [8]. By adding Mn to Al-4.6Mg alloys may produce grain refinement and dispersion strengthening, increasing tensile strength and hardness. The effect of the equal-channel-angular-pressed substrate on the coating formation and anticorrosion performance of the anodized Al–11Si alloy was investigated [9]. The procedure refines both Al and Si phases of the alloy. The optimum coatings of both alloys were obtained after anodization for 30 min. Overall, the coated samples exhibit the best anticorrosion performance, which is evidenced by the concurrently enhanced prevention of coating and improved corrosion resistance of the substrate.

Titanium alloys were another focus of this Special Issue [10,11]: the wires obtained were characterized by high specific strength, good corrosion resistance, high temperature resistance and other excellent comprehensive performance. It has been widely used not only in aerospace, shipbuilding and other high-tech fields, but also increasingly in medical equipment, food safety and other fields [10]. Because titanium alloy wire is relatively difficult to process, it has a large deformation resistance, good elasticity, high flexion ratio and more serious rebound. Additionally, the effect of the different surface treatment methods on the strength of Ti6Al4V titanium alloy sheet adhesive joints was investigated [11]. The following surface treatment methods were used: alkaline degreasing, anodizing, vibratory shot peening, and anodizing with vibrational shot peening. Many investigations were carried out during the experiment: surface roughness measurements, microhardness measurements and strength tests of single-lap adhesive joints fabricated with the use of two epoxy adhesives. It has been found that the application of anodizing followed by vibratory shot peening leads to increased strength of adhesive joints, irrespective of the type of applied epoxy adhesive.

An interesting approach was on amorphous compounds within applications in materials and civil engineering. The Fe_61+x_Co_10−x_W_1_Y_8_B_20_ alloys (where x = 0, 1 or 2) were produced using two production methods with similar cooling rates: injection casting and suction casting [12]. The samples produced were subjected to investigate structure and magnetic properties. The structure of the materials was examined using X-ray diffraction and the magnetic properties of alloys were tested using a vibrating sample magnetometer and a Faraday magnetic balance. It is found that the grain crystallite sizes of the crystalline phases have a decisive influence on the value of the coercive field (especially in the case of hard magnetic phases). It has been shown that privileged areas can already be created during the production process. Their presence determines the crystallization process.

A different approach was discussed in order to evaluate some geopolymers [13], such as structures based on hydrated aluminosilicates units of SiO_4_ and AlO_4_. These units, known as poly(sialate), poly(sialate)-siloxo or poly(sialate)-disiloxo are chemically balanced by the group I cations of K^+^, Li^+^, or Na^+^. Simultaneously, the chemical reaction of formation, known as geopolymerization, governs the orientation of the unit, generating mesoporous structures. Multiple methods were used for pore structure and porosity characterization. The research implements transverse relaxation measurements to monitor the influence introduced by the curing time on the residual liquid phase of geopolymers prepared with two different types of reinforcing particles. According to the obtained results, the geopolymers contain three types of pores formed by the arrangement of the OH^-^ and Si groups (Si-OH), Si-O-Si groups, Si-O-Al groups, and Si-O rings.

All this published research will offer a new approach for further studies in order to create a sustainable society based on knowledge.
